# An Overview on Current Non-invasive Diagnostic Devices in Oral Oncology

**DOI:** 10.3389/fphys.2018.01510

**Published:** 2018-10-25

**Authors:** Marco Mascitti, Giovanna Orsini, Vincenzo Tosco, Riccardo Monterubbianesi, Andrea Balercia, Angelo Putignano, Maurizio Procaccini, Andrea Santarelli

**Affiliations:** ^1^Department of Clinical Sciences and Stomatology, Marche Polytechnic University, Ancona, Italy; ^2^Department of Oral and Head-Neck Surgery, Umberto I General Hospital, Marche Polytechnic University, Ancona, Italy; ^3^National Institute of Health and Science of Aging, INRCA, Ancona, Italy

**Keywords:** light-based detection system, early diagnosis, OSCC, chemiluminescence, autofluorescence, nanotechnology

## Abstract

Oral squamous cell carcinoma (OSCC) is the most common head and neck malignancy, and despite advances in cancer therapies, the overall 5-year survival rate has remained below 50% over the past decades. OSCC is typically preceded by potentially malignant disorders (PMD), but distinguishing high-risk from low-risk PMD is challenging. In the last years, several diagnostic methods as light-based detection systems (LBDS) have been proposed to facilitate the detection of OSCC and PMD. Furthermore, the recent evolution of nanotechnology may provide new opportunities to detect PMD and OSCC at an early stage. Indeed, several preclinical studies showed the potential of nanotechnology to enhance diagnostic accuracy. For these reasons, it is fundamental to conduct studies to evaluate the efficacy of nanotechnology implementation in LBDS. The aim of this article is to review the current literature on LBDS and to provide a summary of the sensitivity and specificity of each technique, and possible future applications of nanotechnologies. The LBDS showed great potential for screening and monitoring oral lesions, but there are several factors that hinder an extensive use of these devices. These devices seem to be useful in assessing lesion margins that must be biopsied. However, to date, conventional oral examination, and tissue biopsy remain the gold standard for OSCC diagnosis. The use of nanotechnologies could be the next step in the evolution of LBDS, thus providing devices that can help clinicians to detect and better monitor oral lesions.

## Introduction

Oral squamous cell carcinoma (OSCC) is the most common head and neck malignancy and the sixth most common tumour worldwide (Warnakulasuriya, 2009). Despite advances in therapies, the overall 5-year survival rate has remained unchanged during the past decades, mainly due to delayed diagnosis (Gomez et al., 2009). OSCC is typically preceded by potentially malignant disorders (PMD), a group of clinically suspicious lesions. Although the majority of PMD do not progress to OSCC, distinguishing high-risk PMD from low-risk PMD is challenging for dental practitioners (Yang et al., 2018). Furthermore, patients treated for OSCC are at risk of developing recurrences and secondary primary tumours, due to field cancerization and/or incomplete surgery (Day and Blot, 1992). Currently, conventional oral examination (COE), consisting in visual and tactile assessment of accessible oral structures, followed by tissue biopsy still constitutes the gold standard for diagnosis of PMD and OSCC. However, there are some limitations of this procedure, such as sampling bias that can lead to underdiagnosis or misdiagnosis, particularly in multifocal lesions (Yang et al., 2018).

The possibility of making an early diagnosis is crucial for reducing high mortality rate and morbidity of OSCC patients. In the last years, several light-based detection systems (LBDS), based on optical properties of biological tissues, have emerged with claims of enhancing oral mucosal examinations and facilitating the detection of PMD and OSCC.

Furthermore, the recent evolution of nanotechnology may provide new opportunities to detect PMD and OSCC at an early stage (El-Sayed et al., 2005). Several preclinical studies showed the potential to enhance diagnostic accuracy of optical diagnostic technologies (e.g., Raman spectroscopy) or imaging techniques (e.g., Magnetic resonance imaging) (Chen et al., 2018). Among the latter techniques, reflectance confocal microscopy seems to improve the evaluation of oral lesions, by detecting backscattered light from illuminated tissue, producing high resolution tissue map. However, several technological limitations need to be resolved to validate diagnostic accuracy (Lucchese et al., 2016). LBDS showed several advantages compared to the aforementioned approaches, such as low cost and ease of use. For these reasons, it is fundamental to conduct studies to evaluate the efficacy of nanotechnology implementation in LBDS.

The aim of this article is to review the literature on LBDS currently on the market (Tables 1, 2), providing clinicians with a better understanding of their advantages and limits, and possible future applications of nanotechnologies.

**Table 1:** Published studies on VELscope^®^ for clinical detection of oral lesions.

Author and year	Patients	Type of lesion	Sens	Spec	PPV	NPV
Poh et al., 2006	20	OSCC	95%	–	100%	–
Lane et al., 2006	44	PMD, OSCC	98%	100%	100%	86%
Roblyer et al., 2009	65	OL	95.9%	96.2%	–	–
Jayaprakash et al., 2009	60	OL	72%	50%	76%	46%
Mehrotra et al., 2010	156	OL	50%	38.9%	6.4%	90.3%
Koch et al., 2011	78	PMD, OSCC	94%	16%	45%	77%
Paderni et al., 2011	175	PMD, OSCC	80.7%	97.5%	93.9%	91.3%
Awan et al., 2011a	126	PMD	84.1%	15.3%	37.8%	61.1%
Scheer et al., 2011	64	OSCC	100%	80.8%	54.5%	100%
Marzouki et al., 2012	85	OL	92%	77%	–	–
McNamara et al., 2012	130	OL	66.7%	6.0%	4.1%	75%
Farah et al., 2012	112	PMD	30%	63%	19%	75%
Rana et al., 2012	123	PMD	100%	74%	16.7%	100%
Hanken et al., 2013	60	PMD	97.9%	33.3%	85.5%	80%
Sawan and Mashlah, 2015	71	OL	100%	74.1%	46.4%	100%
Kaur and Jacobs, 2015	130	OL	67%	62%	29.8%	89%
Jane-Salas et al., 2015	60	OL	40%	80%	62.5%	66.7%
Elvers et al., 2015	20	PMD	–	–	–	–
Ayoub et al., 2015	30	Screening	–	–	–	–
Ohnishi et al., 2016	20	OSCC	91%	100%	100%	58.3%
Scheer et al., 2016	41	OSCC	33.3%	88.6%	33.3%	88.6%
Yamamoto et al., 2017	62	PMD, OSCC	85.9%	26.7%	83.3%	30.8%
Ganga et al., 2017	200	OL	76%	66.3%	24.4%	95.1%
Burian et al., 2017	90	OSCC	–	–	–	–
Huang et al., 2017	140	PMD, OSCC	98.3%	77.6%	91.7%	93.8%
Amirchaghmaghi et al., 2018	21	PMD, OSCC	90%	15%	40%	71%
Farah et al., 2018	11	PMD	–	–	–	–
Canjau et al., 2018	18	OL	94.4%	100%	100%	50%

*OL, oral lesions; PMD, potentially malignant disorders; OSCC, oral squamous cell carcinoma; Sens, sensitivity; Spec, specificity; PPV, positive predictive value; NPV, negative predictive value. Where possible, missing data were recalculated*.

**Table 2:** Published studies on light-based detection systems other than VELscope^®^ for clinical detection of oral lesions.

Author and year	Device	Patients	Type of lesion	Sens	Spec	PPV	NPV
Huber et al., 2004	ViziLite	150	PMD	–	–	–	–
Ram and Siar, 2005	ViziLite	40	PMD, OSCC	100%	14.3%	80%	100%
Epstein et al., 2006	ViziLite	134	PMD	–	–	–	–
Kerr et al., 2006	ViziLite	501	OL	–	–	–	–
Farah and McCullough, 2007	ViziLite	55	OL	100%	0%	18.2%	–
Oh and Laskin, 2007	ViziLite	100	Screening	–	–	–	–
Epstein et al., 2008	ViziLite	84	PMD	100%	55%	37%	100%
Mehrotra et al., 2010	ViziLite	102	OL	0%	75.5%	0%	94.8%
Awan et al., 2011b	ViziLite	126	OL	87%	24%	15%	92%
Mojsa et al., 2012	ViziLite	30	PMD	57.6%	37.5%	79.2%	17.6%
Rajmohan et al., 2012	ViziLite	30	PMD, OSCC	85%	100%	100%	76.9%
Ujaoney et al., 2012	ViziLite	44	PMD	59%	78%	–	–
Vashisht et al., 2014	ViziLite	60	PMD, OSCC	95.5%	84.6%	91.3%	91.7%
Kammerer et al., 2015	ViziLite	44	PMD	100%	30%	26%	100%
Chaudhry et al., 2016	ViziLite	100	PMD	84.8%	41.2%	58.3%	70%
Sweeny et al., 2011	Identafi	88	PMD (white)	50%	98%	50%	98%
			(violet)	50%	81%	11%	97%
			(green)	0%	86%	0%	95%
Lane et al., 2012	Identafi	124	PMD	82%	87%	–	–
Messadi et al., 2014	Identafi	21	OL	–	–	–	–
Lalla et al., 2015	Identafi	342	screening	–	–	–	–
Lalla et al., 2016	Identafi	88	OL (white)	100%	100%	100%	100%
			(Violet)	27.5%	96.3%	61.1%	86.4%
			(Green)	40%	71.7%	22.9%	85.1%
McIntosh et al., 2009	Microlux/DL	50	PMD	77.8%	70.7%	36.8%	93.5%
Ibrahim et al., 2014	Microlux/DL	599	Screening	100%	32.4%	17.9%	100%
Moro et al., 2010	GOCCLES	32	PMD, OSCC	100%	93%	92%	100%
Moro et al., 2015	GOCCLES	61	PMD	96.9%	3.1%	50%	50%

*OL, oral lesions; PMD, potentially malignant disorders; OSCC, oral squamous cell carcinoma; Sens, sensitivity; Spec, specificity; PPV, positive predictive value; NPV, negative predictive value. Where possible, missing data were recalculated*.

## ViziLite^®^

ViziLite^®^ (Zila Pharmaceuticals, Phoenix, AZ, United States) is a chemiluminescence-based detection device designed to facilitate the early identification of PMD and OSCC. In 2002 ViziLite^®^ became the first device approved by FDA for this purpose (Oh and Laskin, 2007). This is a disposable capsule formed by an outer shell of flexible plastic containing acetyl salicylic acid and an inner glass vial containing hydrogen peroxide. To activate it, the capsule is bent to break the inner glass vial, triggering the reaction of the chemicals contained in the two compartments. Consequently, a bluish-white light (430–580 nm) is produced, lasting for 10 min (Liu et al., 2016). A modified version (ViziLite^®^ PLUS) consists of a combination of chemiluminescence and toluidine blue (TB) marking system, an acidophilic dye that selectively stains acidic substances such as DNA. Furthermore, an accessory eyewear has been developed, to allow better isolation of chemiluminescent light (Sambandham et al., 2013). Its clinical use requires a 1-min rinse of 1% acetic acid solution, to desiccate oral tissues, followed by oral examination with 430–580 nm wavelength light. The altered epithelial cells, due to higher nuclear/cytoplasmic ratio, reflect the light and cause the appearance of an “aceto-white” lesion, whereas normal cells appear blue (Nagi et al., 2016).

The first studies regarding ViziLite^®^, published in 2004–2007, were conducted on subjects with different clinical conditions, ranging from normal mucosa to diagnosed OSCC, with the aim to explore the diagnostic utility of chemiluminescence-based strategies (Table 2). In the first reported study, ViziLite^®^ identified a subclinical lesion, suggesting its utility in identifying occult epithelial abnormalities (Huber et al., 2004). In a small cohort of patients with oral lesions, ViziLite^®^ appears to be a better diagnostic tool than TB in detection of OSCC and PMD (Ram and Siar, 2005). Another study highlights the ability of ViziLite^®^ to show brighter and better demarcated lesions than using incandescent light, aiming to enhance the identification of lesions that could be biopsied (Epstein et al., 2006). Unfortunately, these results have not been confirmed, which failed to demonstrate significant improvement in identification and evaluation of oral lesions (Farah and McCullough, 2007; Oh and Laskin, 2007). Interestingly, a cross-sectional study compared ViziLite^®^ and VELscope^®^ to evaluate their clinical utility in diagnosing oral lesions, but the authors failed to demonstrate any superiority to COE (Mehrotra et al., 2010).

For this reason, a new version of this device has been developed (ViziLite^®^ PLUS), aiming to improve the diagnostic power of TB marking system. First results were encouraging, showing that TB reduced the number of false positive cases leaving the false negative rate unchanged (Epstein et al., 2008). On the contrary, ViziLite^®^ PLUS does not seems to be useful to detect malignancies in patients with clearly visible lesions (Mojsa et al., 2012). In fact, some authors described the better diagnostic accuracy of ViziLite^®^ with respect to TB staining alone (Rajmohan et al., 2012; Vashisht et al., 2014), justifying the combined use of these two techniques.

Recently, the results of a clinical study suggested that, although the adjunct of TB to ViziLite^®^ reduced the false positive cases without increasing the number of false negatives, there are little benefits in using this device in general dental practise (Chaudhry et al., 2016).

In conclusion, despite the fact that ViziLite^®^ facilitates the identification of hyperkeratotic areas and may increase the visibility of mucosal lesions, the main limitation is currently the high proportion of false positive and false negative tests, regarding the identification of dysplastic areas rather than hyperkeratosis (Chhabra et al., 2015).

## Velscope^®^

VELscope^®^ (LED Medical Diagnostics, White Rock, BC, Canada) is a hand-held non-magnifying device for direct visualisation of oral mucosa autofluorescence that became commercially available after FDA approval in 2006 (Ayoub et al., 2015). No need of technical measures, such as the use of dimmed light, pre-rinse or lesion-marking solutions, make VELscope^®^ easy to use. It uses a 120 W arc-lamp and a series of philtres and reflectors optimised for producing 400–460 nm wavelength light. The light emitted reaches oral mucosa and excites endogenous autofluorescence substances, called fluorophores (Yamamoto et al., 2017). Preliminary studies, regarding small groups of patients, gave encouraging results (Table 1). In the first reported study, 44 patients with confirmed oral dysplasia or OSCC were evaluated with both COE and VELscope^®^. The results showed that the device can differentiate PMD and OSCC from normal oral mucosa, with high sensitivity and specificity levels (Lane et al., 2006). These results were confirmed in a small OSCC cohort study, in which the use of autofluorescence-guided examination was able to identify subclinical high-risk fields with cancerous changes (Poh et al., 2006). In a study conducted on 60 patients using a semi-quantitative grading system for autofluorescence, VELscope^®^ demonstrate good sensitivity and a better ability to recognise high-grade lesions than COE (Jayaprakash et al., 2009). Another study evaluated 65 subjects with VELscope^®^, using a specific algorithm based on the ratio of red-to-green fluorescence. The authors found that 405 nm wavelength light was able to discriminate neoplastic and non-neoplastic tissue with high sensitivity and specificity (Roblyer et al., 2009).

In a cross-sectional study, 175 patients with at least one clinical lesion were evaluated using VELscope^®^. However, despite the good results, the authors warned that this device could lead to overdiagnosis if used by non-specialists (Paderni et al., 2011). In fact, in the following years several studies on patients with PMD or OSCC reported low specificity values, highlighting this as the primary limitation of VELscope^®^ (Awan et al., 2011a; Koch et al., 2011; Scheer et al., 2011). For these reason, other authors concluded that VELscope^®^ examination alone does not provide significant diagnostic benefit beyond COE in screening for PMD and OSCC, also due to interobserver variability (Farah et al., 2012; McNamara et al., 2012). These results were confirmed by a study on 200 patients, limiting the utility of autofluorescence for OSCC screening (Ganga et al., 2017).

One effort to overcome these shortcomings consists of adding the VELscope^®^ exam to the COE. Indeed, as reported by several authors, the combination of COE and VELscope^®^ examination in patients with oral lesions could provide a significative diagnostic yield (Marzouki et al., 2012; Rana et al., 2012; Hanken et al., 2013). However, these results must be interpreted carefully due to the different inclusion and exclusion criteria used to select the patient cohorts, which can influence both sensitivity and specificity.

Other studies focused on combining VELscope^®^ and other diagnostic tests, aiming to find out better approaches to improve detection of PMD and OSCC. For example, the combination approaches of tissue autofluorescence and salivary protoporphyrin IX levels seems to be effective to distinguish between normal mucosa and high-risk lesions (Kaur and Jacobs, 2015). The use of quantitative analysis of autofluorescence were developed to solve the problem of interobserver variability. Novel methods such as quadratic discriminant analysis or luminance ratio were promising, showing a strong concordance with histopathological diagnosis (Huang et al., 2017; Yamamoto et al., 2017). Recently, a retrospective study based on oral photograph was conducted to find colour distribution patterns related to neoplastic lesions. The fluorescence analysis showed differences in the red-to-green ratios of neoplastic areas, suggesting its clinical utility to detect early OSCC (Burian et al., 2017).

Recently, tissue autofluorescence was used to investigate biological aspects of oral carcinogenesis. In the first *in vitro* study, VELscope^®^ was used to investigate the autofluorescence in a rat tongue carcinogenesis model. The results showed significant changes in autofluorescence pattern during progression to dysplasia and carcinoma (Ohnishi et al., 2016). In another study, RNA sequencing technique was used to identify molecular differences related to autofluorescence patterns. Results were encouraging, demonstrating that the autofluorescence-based excision was successful in achieving a clear molecular margin when excising PMD (Farah et al., 2018). These results confirmed those previously reported in literature, in which VELscope^®^ demonstrated that the actual sizes of some lesions are significantly larger than they look clinically (Elvers et al., 2015).

In conclusion, several criticisms have been made about VELscope^®^, mainly focused to the limited capacity to extend the use of this device in general dental practise. Future research directions are aimed at improving the specificity of this device, allowing wider clinical use of VELscope^®^ in routine general practise (Bhatia et al., 2013).

## Identafi^®^

Identafi^®^ (StarDental - DentalEZ, Lancaster, PA, United States) is a probe-like device designed for multispectral screening of PMD, approved by FDA in 2009 as oral screening device (Vigneswaran et al., 2009). Identafi^®^ has three light sources of different wavelengths: white, violet (405 nm), and green-amber (545 nm) lights, that can be sequentially used in oral examination. While white light provides classical visualisation of oral mucosa, violet light excites endogen fluorophores, enabling the assessment of mucosa autofluorescence, like VELscope^®^. Green-amber light, through the reflectance spectroscopy, excites haemoglobin molecules in the blood, with the aim to visualise the vasculature (Messadi et al., 2014). A mirror is attached to the probe to help visualise relatively obscure areas in oral cavity.

The first clinical trial with Identafi^®^ was conducted on 88 patients who were treated previously for OSCC (Table 2). Screening results with white, violet, and green lights were compared to each other, showing limited benefits of tissue reflectance and autofluorescence in detecting high-risk lesions (Sweeny et al., 2011). In 2012, was reported a case series of PMD patients with the aim to evaluate the efficacy of Identafi^®^. Although the results are not clearly described, this device seems to be helpful in identifying characteristics not otherwise visible to the COE (Lane et al., 2012).

In a pilot study, Identafi^®^ was used to evaluate tissue vascularity of PMD and to compare with the histological grading of the lesions using a vascular marker (CD34). The results found a correlation between tissue reflectance and histological assessment of vascular structure, in both OSCC and non-cancerous lesions (Messadi et al., 2014).

Two studies on the effectiveness of Identafi^®^ were conducted on Australian population. In the first one, 342 urban Indigenous community members were screened for oral lesions using reflectance spectroscopy and autofluorescence imaging. Identafi^®^ improved the visibility of oral cavity lesions and was capable to find new lesions not seen during COE, although the prevalence of oral pigmentation in this community could hamper the use of autofluorescence screening systems (Lalla et al., 2015). In the second study, 88 patients were evaluated with Identafi^®^, showing good specificity, negative predictive value, and accuracy (Lalla et al., 2016).

Taken together, the use of Identafi provide the clinician with more data than COE. Unfortunately, the results interpretation requires high level of experience and clinical training in oral pathology, suggesting that its usage should be limited to reference centres for oral pathology (Lalla et al., 2016).

## Other Devices

Microlux/DL^TM^ (AdDent Inc., Danbury, CT, United States) is a chemiluminescence-based device which became commercially available after FDA approval in 2005. This device has a diffused blue-white LED light source and a fibre optic light guide (McIntosh et al., 2009). It uses the same principles of ViziLite^®^: after 1-min rinse with 1% acetic acid, oral examination is performed with 460–555 nm wavelength light. Altered epithelial cells cause the appearance of “aceto-white” lesions, and LED light source makes the lesion more easily recognisable. Furthermore, the use of TB can be used in conjunction with Microlux/DL^TM^, to enhance the visualisation of dysplastic areas (Ibrahim et al., 2014). In 2009 was conducted a study on 50 patients with oral white lesions to evaluate the efficacy of Microlux/DL^TM^. The results showed that this device can enhance visualisation of oral mucosa, but no clinical improvement was observed, due to poor ability to distinguish between benign and malignant lesions (McIntosh et al., 2009). Another trial that evaluated the effectiveness of Microlux/DL^TM^ was carried out in 2014. 599 patients were examined with COE and Microlux/DL^TM^ with and without TB, showing high sensitivity but low specificity, indicating that this device is not effective to distinguish between benign and malignant lesions, although seems to be a promising screening test for oral lesions (Ibrahim et al., 2014).

GOCCLES^®^ is a medical device (Pierrel S.p.A, Italy) approved by FDA in 2015. This is a low cost and easy-to-use device consisting in a pair of glasses equipped with special optical philtres that allows autofluorescence detection. Indeed, GOCCLES^®^ was created to provide an easy and low cost mean of identification of autofluorescence abnormalities in oral cavity with the use of any dental curing light (Moro et al., 2015). In 2010 was reported the first study on GOCCLES^®^ in a small cohort of selected patients, showing high sensitivity and specificity (Moro et al., 2010). Five years later, a non-randomised multicentre trial was conducted on patients at risk for OSCC, suggesting the need for further researches to define the diagnostic performance of this device (Moro et al., 2015). Indeed, despite the low cost of GOCCLES^®^ could encourage more careful examinations, its main limitation seems to be the interobserver variability, that could be overcome by proper training.

In recent years, other instruments have been developed and commercialised for facilitate the early identification of oral lesions. Their operating principle is equivalent to the devices described above, using either autofluorescence or chemiluminescence detection. However, their clinical effectiveness is currently hampered by the lack of published studies. For these reasons, they will only be mentioned briefly here. Bio/Screen^®^ (AdDent Inc., Danbury, CT, United States), an instrument with five violet (390–430 nm) high-power LED, designed to enhance the visualisation of mucosal abnormalities through the use of tissue autofluorescence (Kahn et al., 2018).

Orascoptic DK^TM^ system (Orascoptic, Middleton, WI, United States) is another chemiluminescence-based device, designed to improve the visualisation of oral lesions through the use of blue-white LED light and oral rinse of 1% acetic acid solution (Patton et al., 2008).

Sapphire^®^ Plus LD (DenMat Holdings, Lompoc, CA, United States), DentLight DOE^TM^ Oral Exam System (DentLight, Richardson, TX, United States), and OralID^TM^ 2.0 (Forward Science Technologies, Stafford, TX, United States) are other tissue autofluorescence-based devices developed in order to detect oral lesions (Kahn et al., 2018).

## Conclusion and Future Perspectives

The diagnostic techniques presented here showed great potential for screening and monitoring oral lesions (Liu et al., 2016). Unfortunately, to date several factors hinder an extensive use of these devices: (1) data do not demonstrate clear superiority of these methods compared to COE; (2) there remains the need for well-designed multicentre prospective studies; (3) these devices exhibit a not-negligible interobserver variability, limiting their use to clinicians with significant experience in oral pathology (Patton et al., 2008; Carreras-Torras and Gay-Escoda, 2015).

However, the current evidence suggests that these devices: (1) seem to be useful in assessing lesion margins that must be biopsied and, therefore, may be useful in surgical management; (2) can be used to investigate biological aspects of oral carcinogenesis, leading to more accurate methods for interpreting data from LBDS; (3) can be enhanced with new approaches used to analyse optical imaging data, with the aim to quantify the results obtained; (4) lowering the costs of these devices could indirectly lead to greater attention for oral lesions among both patients and general dental practitioners, allowing in turn to promote a culture of oral cancer prevention (Carreras-Torras and Gay-Escoda, 2015; Moro et al., 2015); (5) finally, the possibility of implementing LBDS through the use of tissue-marking dyes can in principle allows to develop strategies for the use of nanoparticles. Indeed, nanoparticles can provide molecular targeted imaging, with higher image contrast and resolution. For example, a promising nanotechnology in oral diagnostic research is the quantum dots, consisting in nanometre-sized semiconductor crystals (Walling et al., 2009). The biophysical characteristics of these particles confer several advantages over conventional dyes and fluorescent proteins. The possibility to link the quantum dots to molecules with the ability to target cancer cells make them ideal for diagnostic applications in detecting PMD and OSCC (Chen et al., 2018). Therefore, the use of nanotechnologies could be the next step in the evolution of LBDS, providing devices that can help clinicians to detect and better monitor oral lesions.

## Author Contributions

MM, AS, and MP conceived the literature review. MM and VT described the VELscope^®^ system. AS and RM described the ViziLite^®^ system. AB and GO described the Identafi^®^ system. AP, MP, and GO wrote the concluding remarks. All authors discussed and approved the final version of the manuscript.

## Conflict of Interest Statement

The authors declare that the research was conducted in the absence of any commercial or financial relationships that could be construed as a potential conflict of interest.
